# Supplementary material for: Effect of the Propionylation Method on the Deformability under Thermal Pressure of Block-Shaped Wood
Source: Molecules. 2021 Jun 10;26(12):3539. doi: 10.3390/molecules26123539 (PMC8229580; doi:10.3390/molecules26123539)
Supplement: Supplementary file 1 [file molecules-26-03539-s001.zip › molecules-1240496-supplementary.pdf]

Supplementary Information

Effect of the propionylation method on the deformability under thermal pressure of block-shaped wood

Mitsuru Abe*, Masako Seki, Tsunehisa Miki and Masakazu Nishida

Multi-Materials Research Institute, National Institute of Advanced Industrial Science and Technology (AIST), 2266-98 Shimoshidami, Moriyamaku, Nagoya 463-8560, Japan; m.abe@aist.go.jp

* Correspondence: m.abe@aist.go.jp; Tel.: +81-52-736-7209

Contents:

Figure S1. ^{1}H MAS NMR spectra of the propionylated cypress by the treatment of sulfuric acid.
Figure S2. ^{13}C CP-MAS NMR spectra of the propionylated cypress by the treatment of sulfuric acid.
Figure S3. ^{13}C PST-MAS NMR spectra of the propionylated cypress by the treatment of sulfuric acid.
Figure S4. T_1H values of the propionyl cypresses treated with sulfuric acid: (a) carbohydrates, (b) propionyl group.

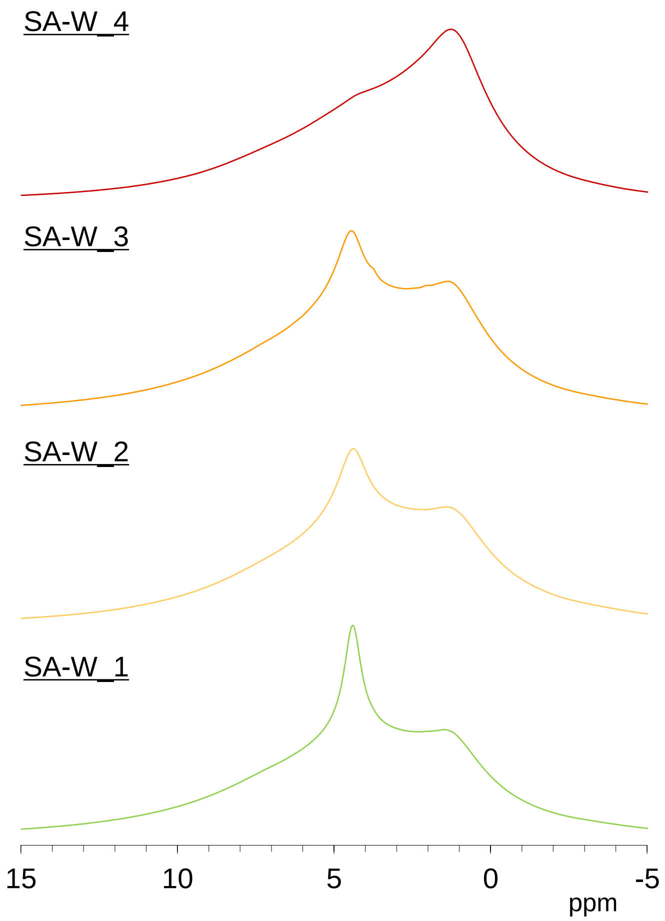

Figure S1. ^{1}H MAS NMR spectra of the propionylated cypress by the treatment of sulfuric acid.

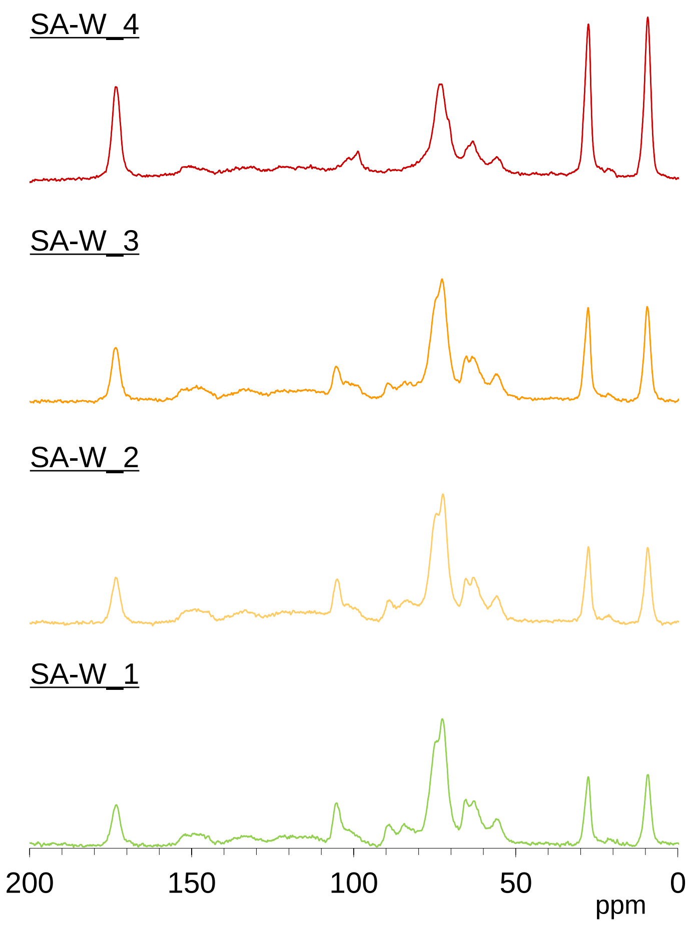

Figure S2. ^{13}C CP-MAS NMR spectra of the propionylated cypress by the treatment of sulfuric acid.

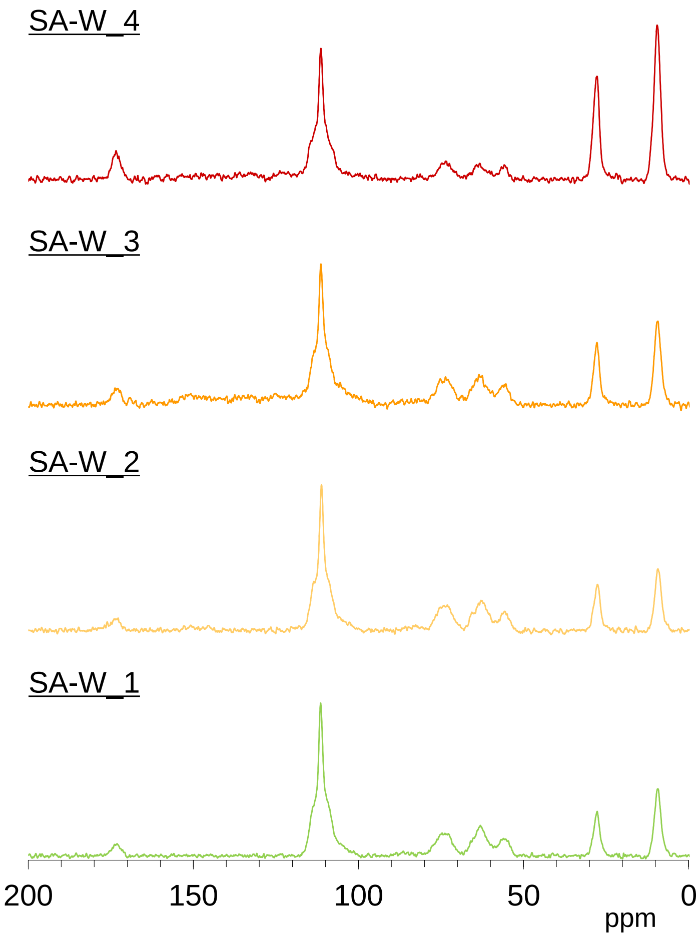

Figure S3. ^{13}C PST-MAS NMR spectra of the propionylated cypress by the treatment of sulfuric acid.

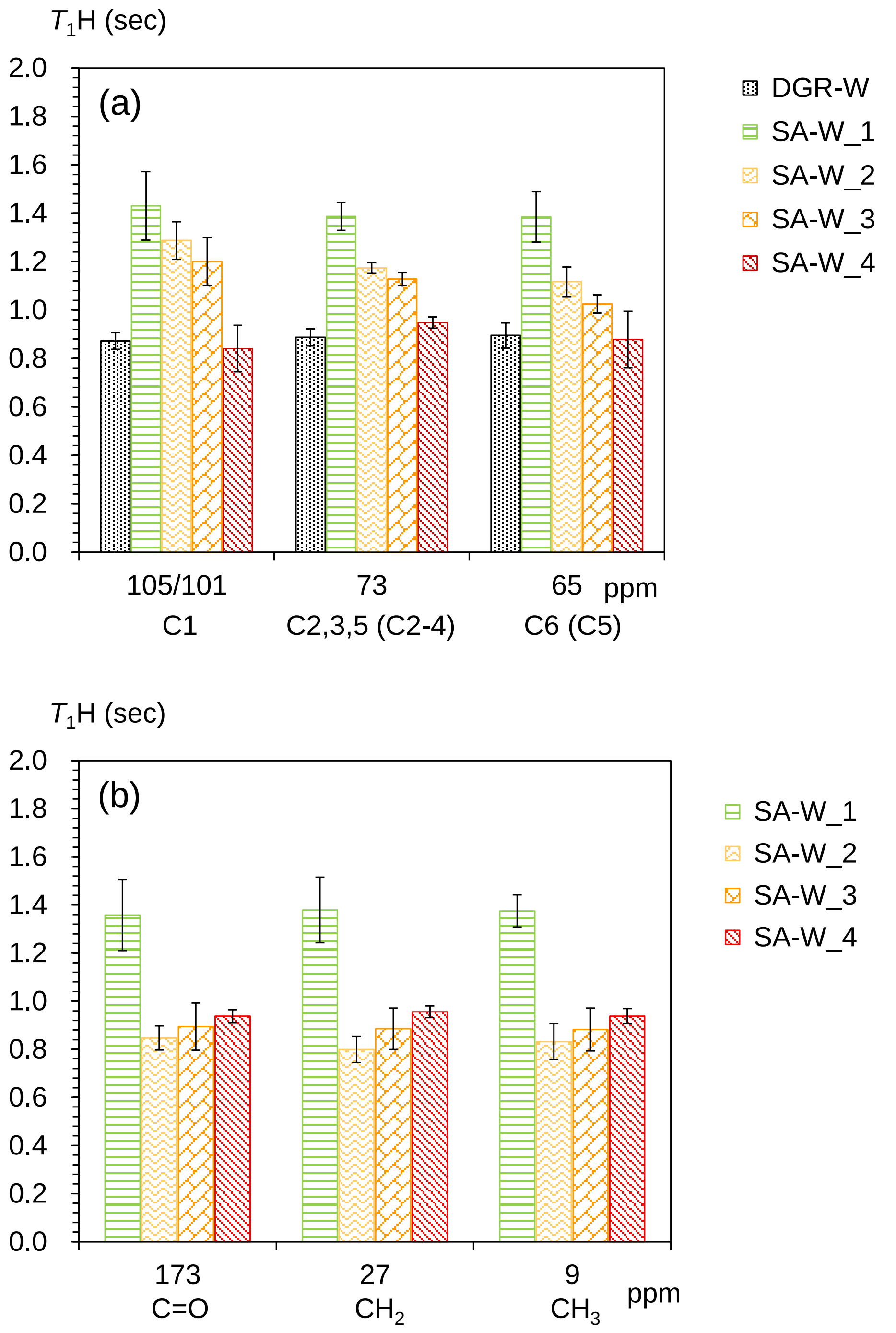

Figure S4. T_1H values of the propionyl cypresses treated with sulfuric acid: (a) carbohydrates, (b) propionyl group.
